# New technologies for diabetes: a review of the present and the future

**DOI:** 10.1186/1687-9856-2012-28

**Published:** 2012-10-26

**Authors:** Neesha Ramchandani, Rubina A Heptulla

**Affiliations:** 1 The Children’s Hospital at Montefiore, Division of Pediatric Endocrinology & Diabetes, 3415 Bainbridge Ave, Bronx, NY, 10467, USA; 2 Albert Einstein College of Medicine, Division of Pediatric Endocrinology, Bronx, NY, 10461, USA

**Keywords:** Diabetes technology, Continuous glucose monitoring, Insulin pump, Blood glucose meter, New therapies, Inhaled insulin, Insulin patch, Insulin pill, SmartInsulin

## Abstract

This review summarizes the technologies in use and in the pipeline for the management of diabetes. The review focuses on glucose meters, continuous glucose monitoring devices, insulin pumps, and getting clinicians connected to technologies. All information presented can be found in the public domain, and was obtained from journal articles, websites, product review tables in patient publications, and professional conferences. The technology concerns, ongoing development and future trends in this area are also discussed.

## 

This review summarizes currently available glucose monitoring and insulin delivery devices and discusses their advantages and disadvantages. It also highlights the “next generation” products under different stages of development. All information was obtained from the public domain.

## I. Blood glucose monitoring devices

Optimal diabetes management relies on accurate glucose monitoring devices. Advances in blood glucose (BG) monitoring technology have resulted in improved accuracy, smaller required blood volumes, and the ability to transfer data between the BG meter and insulin delivery devices.

### A. Capillary blood glucose monitoring

#### Devices

Current home BG meters use capillary blood samples ranging from 0.3-1.5 microliters [1]. The sample is analyzed using either a glucose oxidase or a glucose dehydrogenase reaction. Some strips use the enzymatic/biosensor reaction alone, while others convert the enzymatic reaction into an electrochemical signal first [2]. Although whole blood is used, the meter output is calibrated to provide results that correlate with plasma glucose values. Subtle differences exist among meter performances, such as the ability to withstand temperature extremes and accuracy at higher altitudes. Additionally, certain substances interfere with test strip accuracy. An altered hematocrit will falsely alter the BG in the same direction. Maltose, ascorbate, and acetominaphen interfere with the enzymatic reaction on the test strip, greatly affecting the accuracy of the BG reading [3,4]. A combination of interfering substances can have up to a 193% impact on the accuracy of the BG reading [3]. Despite slight variations in meter advantages, all of the currently FDA approved meters are within 10-15% of actual laboratory plasma glucose values. A reference table of currently available BG meters is published by Diabetes Health every January [1].

##### Enhanced devices

Meter capability is continuously being enhanced. The Freestyle InsuLinx, approved in Europe in 2011, allows patients to program their insulin-to-carbohydrate ratio, correction factor, and target BG into the meter. This information enables the meter to recommend the next insulin dose based on BG and carbohydrate intake [5]. A simplified version of the Freestyle InsuLinx, FDA approved in March 2012 and available for use in the US, works as a meter with a touch screen electronic logbook without bolus calculation capabilities. The One Touch Verio IQ meter, approved in the US and Canada in February 2012, recognizes patterns of hypo- or hyperglycemia and alerts the user to them [6].

##### Cell phone-enhanced meters

The LG KP8400 cell phone add-on has been available in Korea since 2002, and the Infopia LG Glucophone/JVAGO 5965 has been advertised for the US market since 2008. The meter piece is attached to the back of the phone, near the battery pack. Readings are displayed on the cell phone and can be uploaded to an online database [7]. This device is currently unavailable in the US. The European, Bluetooth-enabled GlucoTel system enables wireless transmission of glucose values from a BG meter to a web-based logbook using the individual’s mobile phone as the communication hub [8]. The iBG Star meter and app, recently developed by Sanofi-Aventis, allows certain smartphones (iPhone or iTouch) to serve as the BG meter [9].

##### Plug-and-play devices

The iBG Star meter is one type of plug-and-play device, as the meter must be plugged into a smartphone in order to function. The smartphone serves as this meter’s screen. Another type of plug-and-play device is the Ascensia Contour USB meter, which is a blood glucose meter that also has a USB drive and contains software within it so BG data can be downloaded to a computer without the need of additional software/cables.

##### Combination devices

In the past, Novo Nordisk partnered with Lifescan and created a device called the InDuo, which was the world’s first combined BG meter and insulin delivery device. The meter debuted in 2002, and for undisclosed reasons was discontinued in 2006 [10]. BG meters that double as remote controls for insulin pumps are currently available (see below). After a several year hiatus, BG meter/insulin delivery combination devices are now being revisited [11].

##### Communication with other devices

Radiofrequency technology is used to transfer BG data to insulin pumps; all currently available insulin pumps are enabled with this feature. The Medtronic MiniMed pumps’ associated meters (One Touch UltraLink and Ascensia Contour next link) send BG values to the pump to be incorporated into the calculation of the insulin dose. The Animas One Touch Ping, OmniPod PDM, and Accu-Chek Aviva Combo meters serve as the remote control to the pump itself. The Animas Vibe and Medtronic MiniMed Paradigm systems receive information from their paired real-time continuous glucose monitoring transmitters and display the information on the pump’s screen.

#### Future directions/under development

The newest BG monitoring devices on the market and some of those under development appear in Table 1[5,6,9,12-23].

**Table 1:** Innovative new blood glucose monitors (existing & under development)

** Meter**	** Company**	**FDA/CE status**	**Meter type**	** Outstanding feature(s)/How it works**	** Pros**	** Cons**
***Invasive BG Monitors***						
iBG Star	Sanofi Aventis & AgaMatrix	FDA: approved December 2011 CE: approved Spring 2011	Plug-and-play (hardware), invasive	Meter can be integrated into iPhone or iTouch	Integrated with smartphone	Only integrated with iPhone/iTouch; requires a blood sample
Telcare	Telcare Inc.	FDA: approved August 2011 CE: unknown	Multimedia, invasive	Uses 3G cellular service to communicate with smartphone apps and its own web-based software; gives feedback to user with every BG reading; can re-order supplies through meter	Easy to send data to logs and clinicians, gives feedback to user with every BG, easy to reorder supplies	Requires a blood sample
Freestyle InsuLinx	Abbott	FDA: modified version approved March 2012 CE: approved May 2011	Smart meter, invasive	Meter with bolus calculator within	No need to calculate bolus dose	Requires a blood sample (0.3 μl)
One Touch Verio IQ	Lifescan	FDA: approved Feb. 2012 CE: unknown	Smart meter, invasive	Meter with pattern recognition capabilities. Also has a rechargeable battery.	Prompts user when patterns of hypoglycemia or hyperglycemia occur, so medication adjustments can be made	Requires a blood sample (0.4 μl)
Glucose Monitor Tattoo	Massachusetts Institute of Technology, Northeastern University	FDA & CE: pre-submission	Minimally invasive	Superficial, biocompatible tattoo containing special nanosensors that fluoresce in the presence of glucose	Non-invasive BG monitoring, very small (2 mm), fluorescence reader is iPhone compatible	Tattoo needs to be replaced weekly; brightness not yet correlated to specific BG values; will not be available soon
***Non-Invasive Glucose Monitors***						
GlucoTrack	Integrity Applications	FDA: not yet approved CE: approved 2009	Non-invasive	Uses ultrasound, conductivity, and heat capacity in an ear clip sensor to check BG’s	Non-invasive	Concerns about accuracy in real-life settings
Portable blood glucose meter	Grove Instruments	FDA & CE: unknown	Non-invasive	Uses its patented Optical Bridge technology to read glucose through a finger or earlobe in <20 seconds	Non-invasive	Size of device (company is working on miniaturizing it)
Electronic thumb-pad sensor	Baylor University (Texas)	FDA & CE: pre-submission	Non-invasive	Measures changes in electromagnetic energy waves passed through the skin to detect BG	Non-invasive	Unknown
Near infrared optical spectroscopy	Inlight Solutions	FDA & CE: not yet approved	Non-invasive	Uses near infrared optical spectroscopy and multivariate analysis to measure glucose through the skin	Non-invasive	Unknown
LighTouch Technology	LighTouch	FDA & CE: not yet approved	Non-invasive	Light of a specific color is projected into the user’s fingertip and the different colored light that is reprojected from the fingertip is measured	Non-invasive	Unknown; device has been in development since 1999
I-SugarX	Freedom Meditech	FDA & CE: not yet approved	Non-invasive	Measures the fluorescence of glucose in the aqueous solution of the eye	Non-invasive, quick test	Must shine a light in the eye (for <1 second) to get a glucose reading
Contact lens CGM	University of Washington	FDA & CE: pre-submission	Non-invasive	Contact lens with additional enhancements, including an antenna, measures glucose concentrations in tears and sends the signal to a receiver	Non-invasive	User must have a normal tear concentration; device needs further improvement to work in humans
***Non-Invasive Continuous Glucose Monitors***						
Symphony® tCGM System	Echo Therapeutics	FDA & CE: device in development, studies ongoing	Non-invasive, continuous glucose monitor	Skin is prepped with Prelude device to increase permeability (stratum corneum is removed), and a needle-free, wireless transdermal sensor is placed on the area	Non-invasive; Gives a glucose reading every 1 minute; Designed to use in hospital critical care settings	Concerns about possible side effects
Multisensor Glucose Monitoring System	Biovotion AG	FDA & CE: unknown	Non-invasive, multisensor	Uses optical sensors/impedance spectroscopy to deliver continuous information on glucose variations	Non-invasive	Unknown

Non-invasive glucose monitoring is the goal of the future. Past attempts at non-invasive glucose monitoring failed to reliably separate glucose values from noise without an actual blood sample. Scientific and technological progress have allowed for other, different techniques to be tried, which overcome difficulties faced in the past [24]. Some of the devices under development are truly non-invasive, while others require something to be injected or implanted under the skin to enable glucose detection (Table 1, [15-23]).

#### Connecting meters to clinicians and caregivers

GlucoMON, a system developed by Diabetech in 2006, connects One Touch Ultra BG meters to clinicians and caregivers in real time, using 3G cell phone networks to transmit the data to cell phones, e-mails, and pagers [25,26]. The GlucoTel system wirelessly sends BG’s to a web-based logbook using the patient’s mobile phone as the server [8]. The newly available Telcare system uses 3G cell phone networks to enable two-way communication between the meter and smartphones and its web-based server. Clinicians can access their patients’ information through the website and can customize messages that appear on their patients’ meters every time a BG is checked [13,14]. Other online diabetes management software exists, including Cerner [27], Diasend [28], and Carelink [29]. These systems all require the user to actively upload their devices (instead of data being sent automatically). The reports can be seen by patients and clinicians alike provided the clinician has access to the patient’s report. There are some limitations as to which devices can be uploaded into these programs. Carelink can only be used for the Medtronic MiniMed insulin pumps, glucose sensors, and related BG meters [29]; Cerner and Diasend allow for upload of the Animas insulin pump and most commonly used BG meters [27,28].

In the office, clinicians can download data from BG meters using proprietary software and cables. The programs can be installed on individual computers or networked to several computers, and comprehensive reports can be both printed and saved. If patients use these programs at home, reports can be e-mailed to their clinicians for review.

### B. Continuous glucose monitoring (CGM)

#### Basic CGM

Real-time continuous glucose monitoring (RT-CGM) allows for continuous measurement of interstitial glucose concentrations. A small, enzyme-coated filament (<13 mm in length) inserted subcutaneously detects interstitial glucose through a glucose oxidase reaction. The electrochemical signal is transmitted to a receiver using radiofrequency, where it is converted into a glucose value. The receiver can be an insulin pump (Medtronic Paradigm system, Animas Vibe system) or it can be a stand-alone unit (DexCom, Freestyle Navigator, Medtronic Guardian). The Medtronic’s iPro is a blinded system used by clinicians for diagnostic or research purposes. Sensor glucose values are updated every 1–5 minutes. Sensor electrodes can remain in the body for 3–7 days [30].

RT-CGM systems must be calibrated at regular intervals with a fingerstick BG value, both for accuracy and in order to continue giving the user information. Sensor glucose values are generally within 20% of actual BG values [30-36].

#### Benefits

RT-CGM is clinically useful in identifying post-prandial hyperglycemia, overnight hypoglycemia, hypoglycemia in those with hypoglycemia unawareness, and daily glucose trends. Studies show that patients with type 1 diabetes who use RT-CGM at least 60-70% of the time have significant improvements in glycemic control [37-44]. A recent study found sensor use even 41-60% of the time to have beneficial glycemic effects [45]. Early studies found that patients who are at least 25 years of age experienced the greatest benefits from RT-CGM use [37-41]. This was hypothesized to be due to increased RT-CGM use and better adherence to treatment in adults compared with children and adolescents [40]. Newer studies have shown glycemic benefits of RT-CGM in all age groups [46]. In a sample of pediatric patients, Ramchandani et al. [47] found that although only 21% of all subjects who had a sensor used it continuously, 76% of subjects who owned a sensor believed that using RT-CGM helped them to better manage their diabetes.

#### Disadvantages

The difficulty with RT-CGM is getting patients, especially children and adolescents, to use it on a regular basis for extended periods of time. In many, the frequency of RT-CGM use decreases over time, especially when patients experience frustration with the system. The major reasons cited for disuse of RT-CGM are that patients found it annoying, a hassle, and interfering with their lives. Those who stop using it do so because of problematic equipment, insurance issues, and inaccuracy [47]. These issues need to be addressed before RT-CGM is embraced more universally.

#### Connecting CGM to clinicians

Current CGM devices can be uploaded into device-specific software programs and shared with clinicians either as PDF or Word documents (attached to an e-mail or sent by fax), as web-based reports (Medtronic MiniMed CGM devices only), or during an office visit. Glucose sensors can only be downloaded by proprietary programs, and CGM data fails to integrate with electronic medical record databases.

#### Future directions/under development

The goal of RT-CGM is to develop smaller, more accurate devices. Technologies under consideration include an enhanced transdermal system (Symphony tCGM, [48,49]), multiple optical sensors using impedance spectroscopy (Table 1, [50]), an optical fluorescence fiber-optic biosensor using fluorophore-labeled glucose/galactose binding protein technology [51] which is shown to be >95% accurate in vitro [52], non-enzymatic glucose sensors based on nickel (II) oxide electrospun nanofibers [53] and nickel nanoparticles on carbon nanotubes [54], and a small (3.6 mm x 8.7 mm) continuous osmotic injectable/implantable glucose sensor whose performance is reliable in vitro for up to four weeks [55]. Additionally, intravascular glucose sensors for use in the critical care setting in the hospital are in the works [56]. More accurate RT-CGM devices are needed and have great potential for use both with and without an artificial pancreas.

##### Connecting CGM to insulin delivery, including closed-loop

Some insulin pump companies have already connected glucose sensors to insulin delivery devices in the first steps towards an artificial pancreas. The Medtronic MiniMed Veo pump, available in Europe, Australia, and Canada, suspends basal insulin delivery for two hours if the sensor glucose is below a set level and the individual with diabetes fails to respond or the sensor glucose remains low for a given amount of time. Two insulin pump/glucose sensor devices are currently approved for patient use and many more are currently under development. The Juvenile Diabetes Research Foundation (JDRF)’s proposed steps towards development of the artificial pancreas include: 1) a sensor-augmented pump that will shut off for a specific amount of time when the user does not respond to a low glucose alarm, 2) a hypoglycemia minimizer that adjusts insulin delivery when a low glucose is predicted, 3) a hypoglycemia and hyperglycemia minimizer that will decrease insulin delivery if a low glucose is predicted and increase it if a high glucose (e.g., >200 mg/dl) is predicted, 4) a hybrid closed loop, where the user manually assists the system by bolusing for food intake, 5) a fully automated closed loop with insulin only, and 6) a fully automated multi-hormone closed loop system [57]. Currently, Step 1 is commercially available in Europe, Australia, and Canada (the Medtronic Veo system), and Steps 2–6 exist in research studies.

## II. Insulin delivery devices

### A. Insulin pumps

Insulin pumps are the most physiologic means currently available for insulin delivery. Advancements in technology have transformed what in 1964 was once a large piece of equipment to a small, beeper-sized device that delivers both basal and bolus insulin. User input is essential for a pump to function optimally.

Today’s pumps are available in both wired (with external tubing, e.g., Animas, Medtronic MiniMed, Roche) and wireless (without external tubing, e.g., OmniPod) versions. The wired pumps are attached to the body using an infusion set. The current wireless (patch) pumps are about the size of a small computer mouse, and are attached directly to the body without any tubing in between. A small catheter is inserted subcutaneously from the underside of the patch pump by pressing a button on the remote control. This serves as the patch pump’s infusion set. Today, basal rates can be titrated in as small as 0.025-unit increments, and boluses can be given in 0.05-unit increments. Wired pumps can be detached from the site for showers, water sports, and other activities. Currently available patch pumps remain on the body and are waterproof.

Existing insulin pumps allow the user to enter their carbohydrate intake and their BG, and the pump calculates the insulin dose based on their settings. Medtronic MiniMed and Animas both have sensor-augmented pumps available, wherein interstitial glucose readings are checked every five minutes and displayed on the pump’s screen. The Medtronic MiniMed Veo, which is only available outside of the US, has an auto-shutoff feature if the sensor glucose is low and the user does not respond.

#### Benefits

Many studies have found better glycemic control and/or better quality of life in patients who use insulin pumps compared to those on multiple daily injections (MDI) [58-79]. However, other studies have failed to show improvement in glycemic control compared to MDI [80-84]. Even without improvements in glycemic control, the quality of life benefits offered by insulin pump therapy are worthwhile [85,86]. Insulin pumps are easy to use in insulin-requiring patients of all ages, including infants [74-76].

Sensor-augmented pumps improve glycemic control, but only when the sensor is worn at least 41-60% of the time [39,45]. Both adult and pediatric patients with diabetes have attained metabolic benefits using these devices [46].

#### Disadvantages

The biggest potential downside to insulin pump therapy is being tethered to a device, and the fact that the presence of this device is a constant reminder that one has diabetes. However, few patients discontinue insulin pump therapy because of these issues [87,88]. Rapid onset of ketoacidosis (DKA) can occur within 7–8 hrs with insulin pump therapy if there is an infusion set or insulin delivery malfunction [89,90] because there is no long acting insulin to provide a safety net as with MDI [86,91].

#### Future directions/under development

Recently, there has been an explosion of new products being developed in the insulin pump market (Table 2) [31,92-105]. Companies continuously strive to improve upon existing wired insulin pumps by adding meters that double as full-service remote controls for the pump itself (Roche Accu-Chek Combo), becoming sensor-enhanced with easy-to-read output on the pump’s screen (Animas Vibe + the Dexcom G4 sensor), improving on the pump’s hardware to make it lighter and more failsafe (Spring ADI pump), making pump therapy easier by using pre-filled insulin cartridges (Asante’s Pearl pump), and improving on the size, interface, and usability of the pump (Tandem’s t:slim pump).

**Table 2:** Innovative new insulin pumps (existing & under Development)

** Pump**	** Company**	**FDA/CE status**	** Pump type**	**Reservoir volume**	** Pros**	** Cons**
***Wired Pumps (With Tubing)***						
Accu-Chek Combo	Roche	FDA: approved July 2012 CE: approved late 2009/early 2010	Wired	315 units	Meter as full-service remote control	Text on meter remote is small
Animas Vibe	Animas/Johnson & Johnson	FDA: waiting CE: approved June 2011	Wired, sensor-augmented	200 units	Sensor-augmented pump with Dexcom G4 sensor	Meter remote feature from Animas One Touch Ping does not exist with this pump
ADI pump (1^st^ generation)	Spring/D-Medical	FDA: approved June 2008 CE: approved ~June 2008	Wired	300 units	Lighter and more failsafe	
Spring Zone pump (2^nd^ generation)	Spring/D-Medical	FDA: submitted, waiting CE: approved January 2012	Wired	300 units	Small, light, more failsafe than competitors’ pumps	Proprietary reservoir/infusion set connection
Pearl pump	Asanti Solutions	FDA: approved May 2011 CE: approved April 2011	Wired	300 units	Pre-filled insulin cartridges. Pump designed to simplify diabetes care	Not yet available for use
t:slim	Tandem	FDA: approved Nov. 2011 CE: not yet approved	Wired	300 units	Slim, rechargeable power source, micro-USB connectivity from pump to computer	Rechargeable power source
***Patch Pumps***						
OmniPod sensor-augmented pump	Insulet	FDA: submitted, waiting CE: not yet approved	Patch pump	200 units	Sensor-augmented pump; remote control (PDM) doubles as BG meter; ~40% smaller than current OmniPod pump	Pod adhesive issues
Solo micropump	Medingo/Roche	FDA: approved July 2009 CE: not yet approved	Patch pump	200 units	Detachable, has bolus buttons on the side, reusable/rechargeable parts	
Cellnovo pump	Cellnovo	FDA: not yet approved CE: approved Sept. 2011	Patch pump (with small length of external tubing)		Detachable (?), reusable/rechargeable parts, has a built-in activity sensor; world’s first mobile-connected diabetes management system	
Jewel	Debiotech	FDA: application filed 2010, waiting CE: not yet approved	Patch pump	450 units	Detachable, small footprint on the body, bladder (flexible) insulin reservoir, works with existing mobile phone platforms for remote control	
-----	Medtronic MiniMed	FDA & CE: not yet approved	Patch pump	~200 units		
Freehand	Medsolve Technologies	FDA & CE: not yet approved	Patch pump	300 units		
-----	Spring/D-Medical	FDA & CE: not yet approved	Patch pump			
V-Go	Valeritas	FDA: approved December 2010 CE: approved July 2011	Patch pump	56-76 units	Simple, no remote control	Only pre-set doses allowed for both basal and bolus; basal rate is the same hourly rate throughout the day
Finesse	Calibra	FDA: approved January 2010 CE: no information available	“Simple and elegant insulin delivery device”	200 units	Simple, no remote control, allows for bolus delivery only	Can only bolus in pre-set increments; no basal insulin delivery
Nanopump	Debiotech	FDA & CE: not yet approved	Patch pump/Nanopump		Extremely small	

Until recently, infusion sets have remained more or less unchanged over the past several years. Spring’s newly FDA-approved 90-degree luer-lock infusion set is designed to detect site detachment by occluding the insulin delivery if the site should come out of the body [106].

Many companies are working on patch pumps. The proprietary nature of this information precludes completeness of this list. However, all publicly available information is outlined in Table 2. These pumps strive to improve on existing patch pump technology by being detachable (Roche/Medingo Solo, possibly Cellnovo, Debiotech Jewel, others), adding bolus buttons to the side of the patch pump so insulin can be delivered even without the remote control (Roche/Medingo Solo, others), reducing waste by having reusable or rechargeable parts (Roche/Medingo Solo, Cellnovo, others), having a smaller footprint on the body (Debiotech Jewel, others), changing the dimensions so it can hold larger amounts of insulin (Debiotech Jewel holds 450 units), and working with existing cell phone platforms so that another device does not need to be carried (Debiotech Jewel, others). Some simplistic patch pumps/insulin delivery devices that exist (Valeritas V-Go and Calibra’s Finesse) are aimed more towards patients with type 2 diabetes.

Debiotech is also working on a nanopump, a tiny volumetric pump with a pair of check valves that is integrated into a MEMS chip. “The chip is a stack of three layers bonded together: a silicon on insulator (SOI) plate with micromachined pump-structures and two Pyrex cover plates with through-holes” [104].

### B. Inhaled insulin

Inhaled insulin formulations have been under development for several years, and existing products are able to mimic both first- and second-phase insulin responses [107-111]. Pfizer’s Exubera was available in the US market for approximately six months, but it was unsuccessful and was discontinued [112] due to large, difficult to titrate volumes that needed dispensation by a large device. Other formulations of inhaled insulin are in the works [107,113-116], and their absorption and action profiles are excellent [107-111,113-116]. Limitations of inhaled insulin include difficulty titrating the exact dose needed, potential absorption issues through alveolar and/or buccal membranes, the size of the insulin delivery device itself, and the potential of repeated insulin dosing causing hypertrophy or other problems in the lungs [107,117,118]. Additionally, basal insulin must still be injected despite the use of inhaled insulin.

### C. Insulin pills

Insulin is a protein which is degraded by digestive enzymes and hence must be taken as an injection. However, by using different sized particles, coverings, and inhibitors which allow for insulin to be transported through the gastrointestinal tract, a few companies have developed an insulin pill that is taken by mouth and works like an injection of quick-acting insulin. Limitations of this preparation include that the pill must be taken on an empty stomach for proper absorption. Additionally, if the patient has a second helping of food, the second dose of the insulin pill is not effective because the food that is already in the stomach prevents its absorption [119-123]. Insulin pills are not yet FDA approved.

### D. Insulin patches/Transdermal insulin delivery

Transdermal insulin delivery allows insulin to be absorbed through the skin and can be given anywhere on the body, regardless of the body composition of the person with diabetes. Insulin patches can be potentially used with both rapid-acting and long-acting insulins [124-126]. However, no matter which insulin is put into the patch, all insulin patches have worked like basal insulin until recently [127]. Insulin patches are not yet commercially available.

### E. Smart insulin

Initially a creation of a nanotechnology scientist from MIT and now being developed by Merck, SmartInsulin is an insulin that has been chemically modified to release insulin in response to glucose in the bloodstream. The chemical component attached to the insulin, a biodegradable polymer containing sticky sugar groups, causes it to remain insoluble in the body until a certain concentration of glucose is reached. The glucose then attracts the components that make the insulin insoluble, pulling them away from the hormone and allowing it to become active. It is believed that this will allow for better postprandial insulin coverage while minimizing the incidence of hypoglycemia, all with only one injection per day [128,129]. SmartInsulin is still in preclinical studies.

## Conclusion

We are in the midst of a revolution of technological advancements in diabetes care. This technology boom and associated variety of diabetes management tools enable clinicians to develop new and innovative means of treating their patients. Additionally, these advancements have the potential to decrease the burden of diabetes management on the patients themselves. Advances in diabetes technology will continue to improve patient care and its delivery, and may one day lead to fully automated treatment systems for people with diabetes mellitus.

## Abbreviations

BG: Blood glucose; FDA: Food and Drug Administration; US: United States; PDM: Personal Diabetes Manager; CGM: Continuous glucose monitoring; RT-CGM: Real-time continuous glucose monitoring; JDRF: Juvenile Diabetes Research Foundation; MDI: Multiple daily injections; DKA: Diabetic ketoacidosis; MEMS: Micro-Electro-Mechanical Systems; MIT: Massachusetts Institute of Technology (Cambridge, MA).

## Competing interests

The authors of this article do not have any conflicts of interest to disclose. NR was on the Educator Advisory Panel for Medingo (now a division of Roche) from 9/06 – 6/11, and is currently on the Educator Advisory Boards for both Unomedical and Halozyme. Both authors are working on an NIH-funded project with the Medtronic MiniMed ePID closed loop system.

## Authors’ contributions

NR researched the material, wrote the paper, and created the tables. RAH confirmed the research and edited the manuscript. Both authors read and approved the final manuscript.

## Authors’ informations

Neesha Ramchandani, PNP, CDE is a Pediatric Nurse Practitioner in Diabetes at the Children’s Hospital of Montefiore in the Bronx, NY, USA. Neesha has a strong interest in both diabetes technology and pediatric diabetes. She has published many papers on diabetes technology, including the first paper in the literature on insulin pumps from the time of diagnosis of type 1 diabetes without any additional agent and the first paper in the literature on why people do not use glucose sensors. Neesha has presented her work around the world, including in the United States, many countries in Europe, Turkey, South Africa, and Israel. She is currently involved in research using the closed loop/artificial pancreas system.

Rubina A. Heptulla, MD is the Chief of the Division of Pediatric Endocrinology & Diabetes at the Children’s Hospital of Montefiore and Professor of Medicine at Albert Einstein College of Medicine, both in the Bronx, NY, USA. Dr. Heptulla is the author of numerous publications in the area of pediatric type 1 diabetes and is an NIH funded researcher for many years.
